# Eustachian tube dysfunction in patients with house dust mite-allergic rhinitis

**DOI:** 10.1186/s13601-020-00328-9

**Published:** 2020-07-15

**Authors:** Yun Ma, Maojin Liang, Peng Tian, Xiang Liu, Hua Dang, Qiujian Chen, Hua Zou, Yiqing Zheng

**Affiliations:** Department of Otorhinolaryngology, Sun Yat-sen Memorial Hospital, Sun Yat-sen University, Guangzhou, People’s Republic of China

**Keywords:** Allergic rhinitis, Eustachian tube dysfunction, Nasal obstruction

## Abstract

**Background:**

One of the important pathogeneses of eustachian tube dysfunction (ETD) is nasal inflammatory disease. The prevalence of allergic rhinitis (AR) in adults ranges from 10 to 30% worldwide. However, research on the status of eustachian tubes in AR patients is still very limited.

**Methods:**

This prospective controlled cross-sectional study recruited 59 volunteers and 59 patients with AR from Sun Yat-sen Memorial Hospital. Visual analogue scale (VAS) scores for AR symptoms and seven-item Eustachian Tube Dysfunction Questionnaire (ETDQ-7) scores were collected for both groups. Nasal endoscopy, tympanography and eustachian tube pressure measurement (tubomanometry, TMM) were used for objective assessment. All AR patients underwent 1 month of treatment with mometasone furoate nasal spray and oral loratadine. Then, the nasal condition and eustachian tube status were again evaluated.

**Results:**

TMM examination revealed that 22 patients (39 ears, 33.1%) among the AR patients and 5 healthy controls (7 ears, 5.9%) had abnormal eustachian pressure. Twenty-two AR patients (37.3%) and 9 healthy controls had an ETDQ-7 score ≥ 15. With regard to nasal symptoms of AR, the VAS scores of nasal obstruction were correlated with the ETDQ-7 scores, and the correlation coefficient was r = 0.5124 (*p *< 0.0001). Nasal endoscopic scores were also positively correlated with ETDQ-7 scores, with a correlation coefficient of 0.7328 (*p *< 0.0001). After 1 month of treatment, VAS scores of nasal symptoms, endoscopic scores and ETDQ-7 scores were significantly decreased in AR patients (*p *< 0.0001), and TMM examination also suggested that eustachian tube function was significantly improved after treatment (*p *< 0.0001).

**Conclusions:**

AR patients, especially those with severe nasal obstruction, could have ETD. The local conditions of the pharyngeal orifices of the eustachian tubes are closely related to the symptoms of ETD. After treatment with nasal glucocorticoids and oral antihistamines, eustachian tube function can significantly improve as nasal symptoms subside.

*Trial registration* Chinese Clinical Trial Registery (ChiCTR2000029071) Registered 12 January 2020—Retrospectively registered, http://www.chictr.org.cn/edit.aspx?pid=48328&htm=4

## Background

Eustachian tubes maintain middle ear ventilation and pressure balance, enable mucus clearance and protect the middle ear from pathogens and sounds. Eustachian tube dysfunction (ETD) is estimated to affect 1% of adults and 40% of children [1–3]. Prolonged EDT may cause otitis media effusion (OME), especially in children. Obstructive ETD, the most common subtype, usually results from inflammatory conditions. For example, smoking, chronic rhinosinusitis (CRS), allergic rhinitis (AR) and laryngopharyngeal reflux [4] have been proposed as potential contributing factors affecting eustachian tube function [5]. Some studies have shown that the incidence of ETD in CRS patients is 48.5–68% [6, 7].

AR affects more than 500 million people worldwide [8]. In China, the prevalence of self-reported AR has been reported to be 9.8% in children and 17.6% in adults [9], and it is still rising [10]. According to the severity of symptoms and quality of life, AR could be divided into mild and moderate-severe AR [8]. Moderate-severe AR is more likely to affect the quality of life and social functioning than mild AR [11, 12]. ETD is widely considered to be an important comorbidity associated with AR [13]. However, the nasal symptoms of AR are uncomfortable and might cause patients not to notice ETD symptoms. Lack of proper control of AR for long periods may lead to chronic ETD that necessitates further treatment or even balloon dilation of the eustachian tube (BDET) [14–16]. However, to date, the precise incidence of ET inflammation in AR and the therapeutic efficacy of anti-allergy treatment for ETD have not been well characterized.

The aim of this study was to comprehensively assess eustachian tube function in patients with AR and to determine the main nasal symptoms and local factors that contribute most to ETD.

## Methods

### Study subjects

This study was approved by the Sun Yat-sen Memorial Hospital Institutional Review Board. We recruited consecutive adult AR patients (aged 18–60) who visited Sun Yat-sen Memorial Hospital from June 2019 to January 2020 due to their nasal symptoms. The AR subjects represented the outpatients of ENT clinic in a tertiary hospital. AR was diagnosed according to the criteria of the Initiative on Allergic Rhinitis and its Impact on Asthma (ARIA) [17]. The inclusion criteria included (1) clinical symptoms of AR evidenced by nasal obstruction, itching, sneezing and watery rhinorrhoea; (2) pale nasal mucosa, watery secretions or inferior turbinate hypertrophy; and (3) a positive skin prick test (SPT) for house dust mite or specific immunoglobulin E (sIgE) to *Dermatophagoides pteronyssinus* (*Der p*1). The exclusion criteria were as follows: (1) a history of CRS or rhinitis; (2) a history of chronic otitis media (COM) or otitis media with effusion (OME); (3) a history of acute upper respiratory tract infection (AURT) in the last 4 weeks and (4) a history of glucocorticoid or antihistamine use within the last 4 weeks. Healthy volunteers aged 18–60 were collected at the same time for control group. The inclusion criteria for control group included (1) without any nasal or ear symptoms; (2) no abnormal nose and ear signs were found by endoscopy and (3) SPT excluded allergic diseases. The exclusion criteria were as follows: (1) a history of CRS, rhinitis; (2) a history of COM or OME; (3) a history of AURT in the last 4 weeks and (4) a history of glucocorticoid or antihistamine use within the last 4 weeks. We eventually included 59 AR patients and 59 healthy controls. More details for the definition of AR could be found in Additional file 1: Table S1-2 and Figure S1.

### Evaluation and intervention

**Fig. 1:**
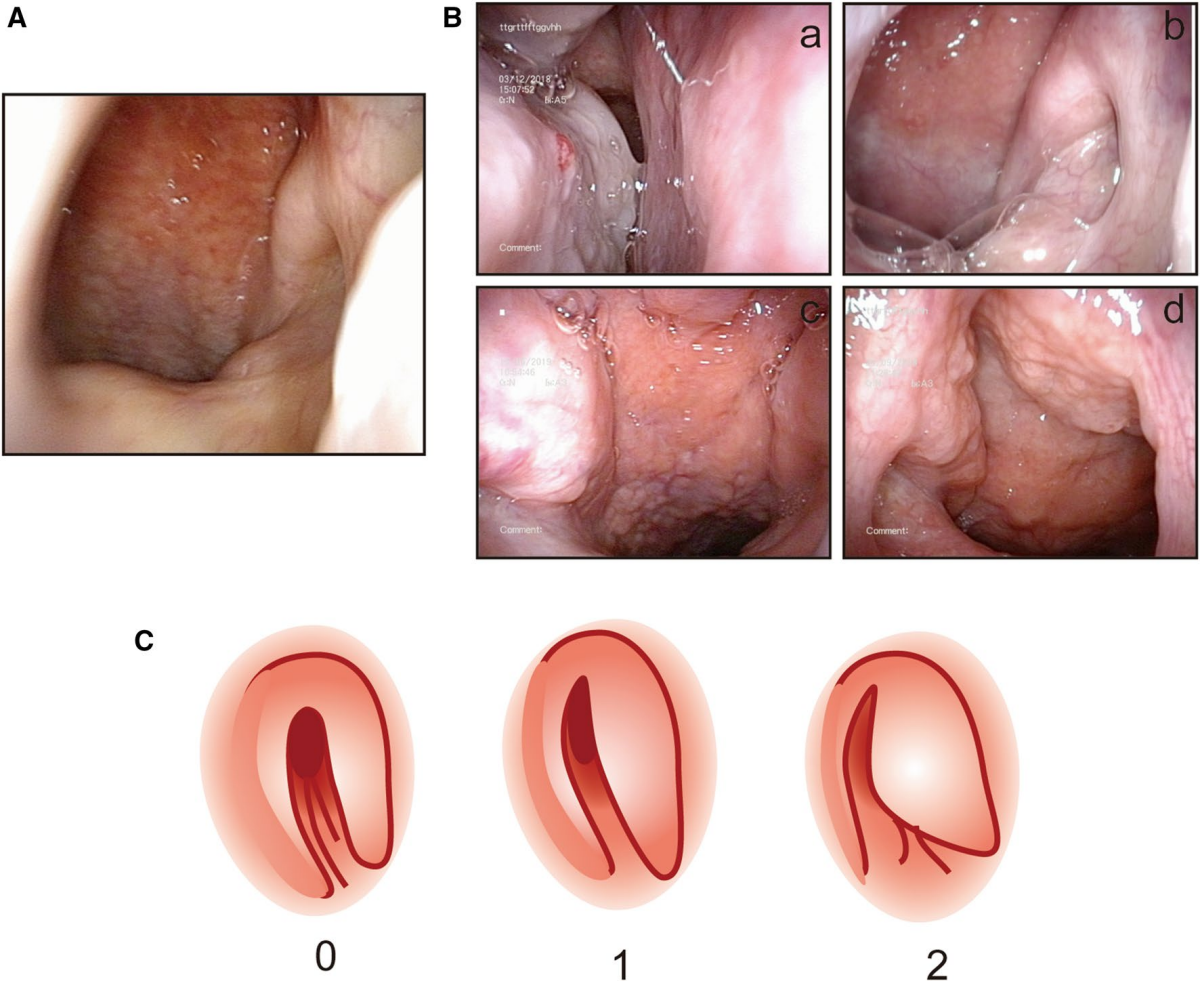
Representative images for nasal endoscopic scores. **A** Representative image of posterior nostril area in control subject. **B** Representative images of posterior turbinate swelling (a), posterior nostril secretion (b), torus tubarius swelling (c), and nasopharyngeal lymphatic hyperplasia (d). **C**  Schematic diagram of the shapes of pharyngeal orifices and its nasal endoscopic score (under 30° nasal endoscope)

All patients were asked to complete a visual analogue scale (VAS) assessment for nasal symptoms, because the VAS score was significantly related to the rhinoconjunctivitis quality of life questionnaire (RQLQ) score and was considered a simple and effective method for assessing AR [18]. The severity of AR was classified as “mild” or “moderate/severe” on the basis of four symptoms (sleep disturbance, impairment of daily activities, leisure and/or sport, impairment of school or work, and troublesome symptoms). We defined as mild AR if all symptoms were absent, moderate/severe AR if there was at least one symptom [8]. The VAS score for each symptom ranged from 0 to 10. The 7-item Eustachian Tube Dysfunction Questionnaire (ETDQ-7) was used for evaluation of ETD symptoms, and the threshold for ETD was considered to be a score of 14.5, as described in a previous study [19]. Then, nasal endoscopy was performed to record the endoscopic score, and acoustic immittance and eustachian tube pressure measurement (tubomanometry, TMM) tests were performed to evaluate eustachian tube function. Tympanogram tracings are classified as type A (normal), type B (suggesting fluid in the middle ear space), and type C (indicating a significantly negative pressure in the middle ear).The Tubomanometry (SPIGGLE & THEIS Medizintechnik GmbH, Germany) was used for eustachian tube pressure measurement. With the application of excess pressure into the nose and rhinopharynx during swallowing, the tubomanometry can record the opening parameters of the Eustachian tube and the pressure equalization function of the middle ear, which reflects the status of eustachian tube at the time of testing. A value of R < 1 in TMM were considered to be normal. The nasal endoscopic scores for the pharyngeal orifices of eustachian tubes ranged from 0 to 20, and the method and representative pictures are shown in Table 1 and Fig. 1. All the AR patients took loratadine 10 mg and mometasone furoate sprayed twice on each nostril daily for 1 month. Other treatments including nasal lavage and nasal decongestants were not allowed in this study. After 1 month of treatment, all the parameters above were assessed again.

**Table 1:** Nasal endoscopic for pharyngeal orifices of eustachian tube

Characteristics	Side	score
Posterior turbinate swelling	Left	0–2
	Right	0–2
Posterior nostril secretion	Left	0–2
	Right	0–2
Torus tubarius swelling	Left	0–2
	Right	0–2
Nasopharyngeal lymphatic hyperplasia	Left	0–2
	Right	0–2
The shape of pharyngeal oridices	Left	0–2
	Right	0–2
Total score		0–20

Posterior turbinate swelling 0 = no swelling, 1 = mild (still has gap with nasal septum) 2 = severe (very close to nasal septum)

Posterior nostril secretion 0 = none 1 = little, watery 2 = attached by viscous secretion

Torus tubarius swelling 0 = none 1 = mild 2 = severe

Nasopharyngeal lymphatic hyperplasia 0 = none 1 = mild 2 = severe

The shape of pharyngeal oridices 0 = round or oval, 1 = fissure, 2 = can’t see

### Statistical analysis

Statistical analysis was performed with GraphPad Prism (ver-sion 7.00 for MacOS, GraphPad Software, La Jolla, San Diego, CA, USA; http://www.graphpad.com). Descriptive analyses were utilized to describe the sample characteristics, and the data are presented as number (%), mean ± standard deviation (SD), or median (inter-quartile range, IQR) according to the tests of normality. The Chi square tests for categorical variables and the paired or unpaired Student’s t tests or Wilcoxon rank sum tests for continuous variables were performed. Spearman or Pearson correlation coefficient analyses were applied to assess the correlation between VAS scores and ETDQ-7 scores, depending on the results of the normality test. Chi square tests were used to compare the categorical data. A *p* value < 0.05 was considered to indicate significance. Considering that the same patient has two ears, our statistical analyses for Tympanometry and TMM tests were performed on the number of ears.

## Results

A total of 59 new patients diagnosed with AR (118 AR ears) and 59 volunteers (118 healthy ears) were included in the study. The clinical characteristics of the two groups were shown in Table 2.

**Table 2:** Clinical characteristics for Control group and AR group

	Control group (n = 59)	AR group (n = 59)	χ^2^	*p*
Gender				*p *= 0.5772
Female/male	35/24	32/27	0.3108	
Age (year), mean ± SD	32.86 ± 11.07	35.03 ± 11.8		*p *= 0.6275
Allergen^a^				
House dust mite (*Der p*1)	0	59	118	*p *< 0.0001
Cockroach	0	10	10.93	*p *= 0.0009
Shrimp	0	9	9.743	*p *= 0.0018
Artemisia	0	3	3.078	*p *= 0.0793
Wheat	0	3	3.078	*p *= 0.0793
Soy	0	3	3.078	*p *= 0.0793
Cat	0	2	2.034	*p *= 0.1538
Other allergic diseases				
Asthma	0	0	–	–
Atopic dermatitis	0	0	–	–
Allergic conjunctivitis	0	4	4.14	*p *= 0.0419
Smoker				
Yes/no	13/46	9/50	0.8939	*p *= 0.3444
Laryngopharyngeal reflux				
Yes/no	5/54	7/52	0.3711	*p *= 0.5424
History of acute otitis media				
Yes/no	3/56	5/54	0.5364	*p *= 0.4639
Family history of ear problems				
Yes/no	4/55	3/56	0.1519	*p *= 0.6968
Family history of allergic disease				
Yes/no	2/57	8/51	3.933	*p *= 0.0473

^a^SPT result≥ + or sIgE level > 0.35 kUA/L can be defined as a positive allergen test

### Prevalence of ETD in patients with AR

The ETDQ-7 was used to evaluate the subjective symptoms of ETD. We found that 22 AR patients (37.3%) had an ETDQ-7 score ≥ 15, significantly more than the 9 patients (15.3%) in the control group (χ^2^ = 7.394, *p *= 0.0065) (Table 3).

**Table 3:** Eustachian tube function for AR group and control group

	Control group (59 subjects)	AR group (59 patients)	χ^2^	*p*
Tympanometry^1^ (236 ears)			15.29	*p *= 0.005
Type A, N (%)	116 (98.3%)	99 (83.9%)		
Type B, N (%)	0	4 (3.4%)		
Type C, N (%)	2 (1.7%)	15 (12.7%)		
TMM^1^ (236 ears)			27.65	*p *< 0.0001
Normal, N (%)	111 (94.1%)	79 (66.9%)		
Abnormal, N (%)	7 (5.9%)	39 (33.1%)		
ETDQ-7 Score^2^ (118 patients)			7.394	*p *= 0.0065
< 15, N (%)	50 (84.7%)	37 (62.7%)		
≥ 15, N (%)	9 (15.3%)	22 (37.3%)		

^1^Statistical analysis was performed by the number of ears

^2^Statistical analysis was performed by the number of patients

*TMM* tubomanometry, *ETDQ-7* seven-item Eustachian Tube Dysfunction Questionnaire

Tympanograms and TMM test results were used as objective indicators of ETD. Our results showed that there were significantly fewer type-A tympanograms in the AR group (52 patients; 99 ears, 83.9%) than in the control group (59 subjects; 116 ears, 98.3%) (χ^2^ = 15.29, *p *= 0.005). Twenty-two AR patients (39 ears, 33.1%) and 6 control subjects (7 ears, 5.9%) had abnormal TMM results, and the difference between groups was statistically significant (χ^2^ = 27.65, *p *< 0.0001) (Table 3).

### Correlation of AR with ETD

**Fig. 2:**
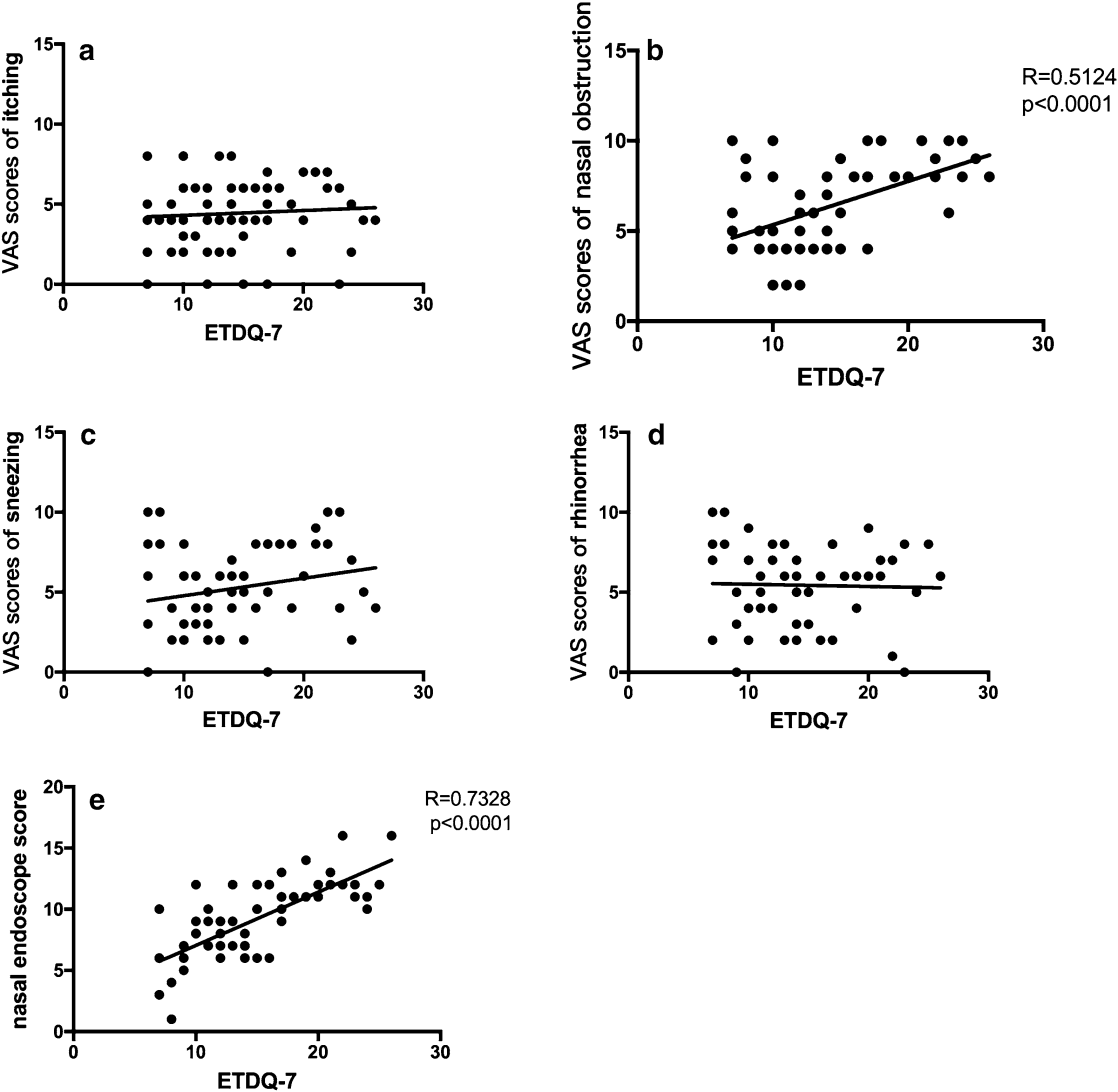
Correlation of nasal symptoms and endoscopic scores with eustachian tube function in HDM-allergic rhinitis patients. **a** The Pearson correlation coefficient between ETDQ-7 and itching VAS scores (normal distribution) was r = 0.0707 (*p *= 0.5947). **b** The Spearman correlation coefficient between ETDQ-7 and nasal obstruction VAS scores (non-normal distribution) was r = 0.5124 (*p *< 0.0001). **c** The Pearson correlation coefficients between ETDQ-7 and sneezing scores (normal distribution) was r = 0.2203 (*p *= 0.0937). **d** The Pearson correlation coefficients between ETDQ-7 and rhinorrhoea scores (normal distribution) was r = − 0.03006 (*p *= 0.8212). **e** The Pearson correlation coefficient between ETDQ-7 and nasal endoscopic scores (normal distribution) was r = 0.7328 (*p *< 0.0001)

The Spearman correlation coefficient was used to evaluate the correlation between the ETDQ-7 and nasal obstruction VAS score (non-normal distribution). A moderate correlation was found between ETDQ-7 scores and nasal obstruction scores (r = 0.5124, *p *< 0.0001). However, other symptoms did not show significant correlations with ETDQ-7 scores. The Pearson correlation coefficients (normal distribution) were as follows: itching, r = 0.0707 (*p *= 0.5947); sneezing, r = 0.2203 (*p *= 0.0937); and rhinorrhoea, r = −0.03006 (*p *= 0.8212). The Pearson correlation coefficient (normal distribution) between ETDQ-7 and nasal endoscopic scores was r = 0.7328 (*p *< 0.0001), suggesting a strong correlation (Fig. 2).

The patients with AR were classified into a mild group (22 patients) and a moderate/severe group (37 patients). We found that there were significantly fewer type A tympanograms in the moderate/severe AR group (27 patients; 57 ears, 77%) than in the mild AR group (22 patients; 42 ears, 95.5%) (χ^2^ = 7.176, *p *= 0.0277). Three mild AR patients (6 ears, 13.6%) and 19 moderate/severe AR patients (33 ears, 44.6%) had abnormal TMM results, and the difference between groups was statistically significant (χ^2^ = 11.95, *p *= 0.0005). With regard to subjective symptoms, significantly more moderate/severe AR patients (19 patients, 51.4%) than mild AR patients (3 patients, 13.7%) had ETDQ-7 scores ≥ 15 (χ^2^ = 8.392, *p *= 0.0038) (Table 4). These data suggest a higher prevalence of ETD in moderate/severe AR patients than in mild AR patients.

**Table 4:** Eustachian tube function for AR severity

	Mild AR (n = 22 patients)	Moderate-severe AR (n = 37 patients)	χ^2^	*p*
Tympanometry^1^ (118 ears)			7.176	*p *= 0.0277
Type A, N (%)	42 (95.5%)	57 (77.0%)		
Type B, N (%)	0	4 (5.4%)		
Type C, N (%)	2 (4.5%)	13 (17.6%)		
TMM^1^ (118 ears)			11.95	*p *= 0.0005
Normal, N (%)	38 (86.4%)	41(55.4%)		
Abnormal, N (%)	6 (13.6%)	33 (44.6%)		
ETDQ-7 Score^2^ (59 patients)			8.392	*p *= 0.0038
< 15, N (%)	19 (86.4%)	18 (48.6%)		
≥ 15, N (%)	3 (13.7%)	19 (51.4%)		

^1^Statistical analysis was performed by the number of ears

^2^Statistical analysis was performed by the number of patients

*TMM* tubomanometry, *ETDQ-7* seven-item Eustachian Tube Dysfunction Questionnaire

**Fig. 3:**
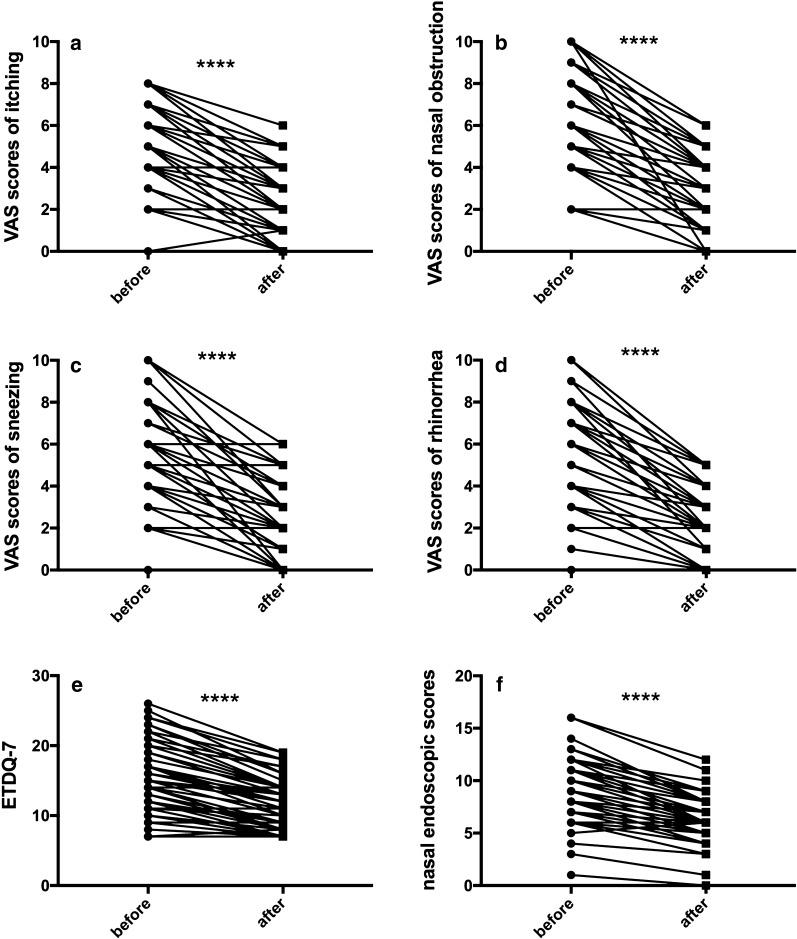
Changes in nasal symptom, ETDQ-7 and endoscopic scores after 1 month of treatment in HDM-allergic rhinitis patients. **a**–**d** The VAS scores for itching, nasal obstruction, sneezing and rhinorrhoea were 4.441 ± 2.199, 6 (4,8), 5.237 ± 2.635 and 5.441 ± 2.437, respectively. After one month of oral antihistamine and nasal mometasone furoate treatment, the VAS scores decreased to 1.915 ± 1.500 for itching, 2.373 ± 1.790 for sneezing, 3 (2,4) for nasal obstruction and 2.424 ± 1.476 for rhinorrhoea (*p *< 0.0001). **e** The ETDQ-7 score decreased from 14.540 ± 5.201 before treatment to 10.900 ± 3.595 after treatment (*p *< 0.0001). **f** The nasal endoscopic score decreased from 9.017 ± 3.088 before treatment to 6.186 ± 2.381 after treatment (*p *< 0.0001)

After 1 month of treatment with loratadine and mometasone furoate, the VAS scores for four major nasal symptoms, the nasal endoscopic scores, and the ETDQ-7 scores were decreased significantly (*p *< 0.0001, Fig. 3). The number of patients with type A tympanograms was increased to 56 patients (109 ears, 92.4%) after treatment, but the difference was not statistically significant (χ^2^ = 5.981, *p *= 0.0503). The prevalence of abnormal TMM was decreased significantly to 10 patients (17 ears, 7.6%) (χ^2^ = 11.33, *p *= 0.0008). The symptoms of ETD were also improved significantly. We found that only 8 AR patients (13.6%) had ETDQ-7 scores ≥ 15 after treatment, significantly fewer than the 22 patients (37.3%) before treatment (χ^2^ = 8.761, *p *= 0.0031) (Table 5). After treatment, ETDQ-7 scores in AR patients showed no significant difference from the control group, but the nasal symptom and endoscopic scores were still higher than control (Additional file 1: Figure S2 and Table S3).

**Table 5:** The changes of eustachian tube after treatment for AR

	Before (n = 59)	After (n = 59)	χ^2^	*p*
Tympanometry^1^ (118 ears)			5.981	*p *= 0.0503
Type A, N (%)	99 (83.9%)	109(92.4%)		
Type B, N (%)	4 (3.4%)	0		
Type C, N (%)	15 (12.7%)	9 (7.6%)		
TMM^1^ (118 ears)			11.33	*p *= 0.0008
Normal, N (%)	79 (66.9%)	101 (85.6%)		
Abnormal, N (%)	39 (33.1%)	17 (14.4%)		
ETDQ-7 Score^2^ (59 patients)			8.761	*p *= 0.0031
< 15, N (%)	37 (62.7%)	51 (86.4%)		
≥ 15, N (%)	22 (37.3%)	8 (13.6%)		

^1^Statistical analysis was performed by the number of ears

^2^Statistical analysis was performed by the number of patients

*TMM* tubomanometry, *ETDQ-7* seven-item Eustachian Tube Dysfunction Questionnaire

**Fig. 4:**
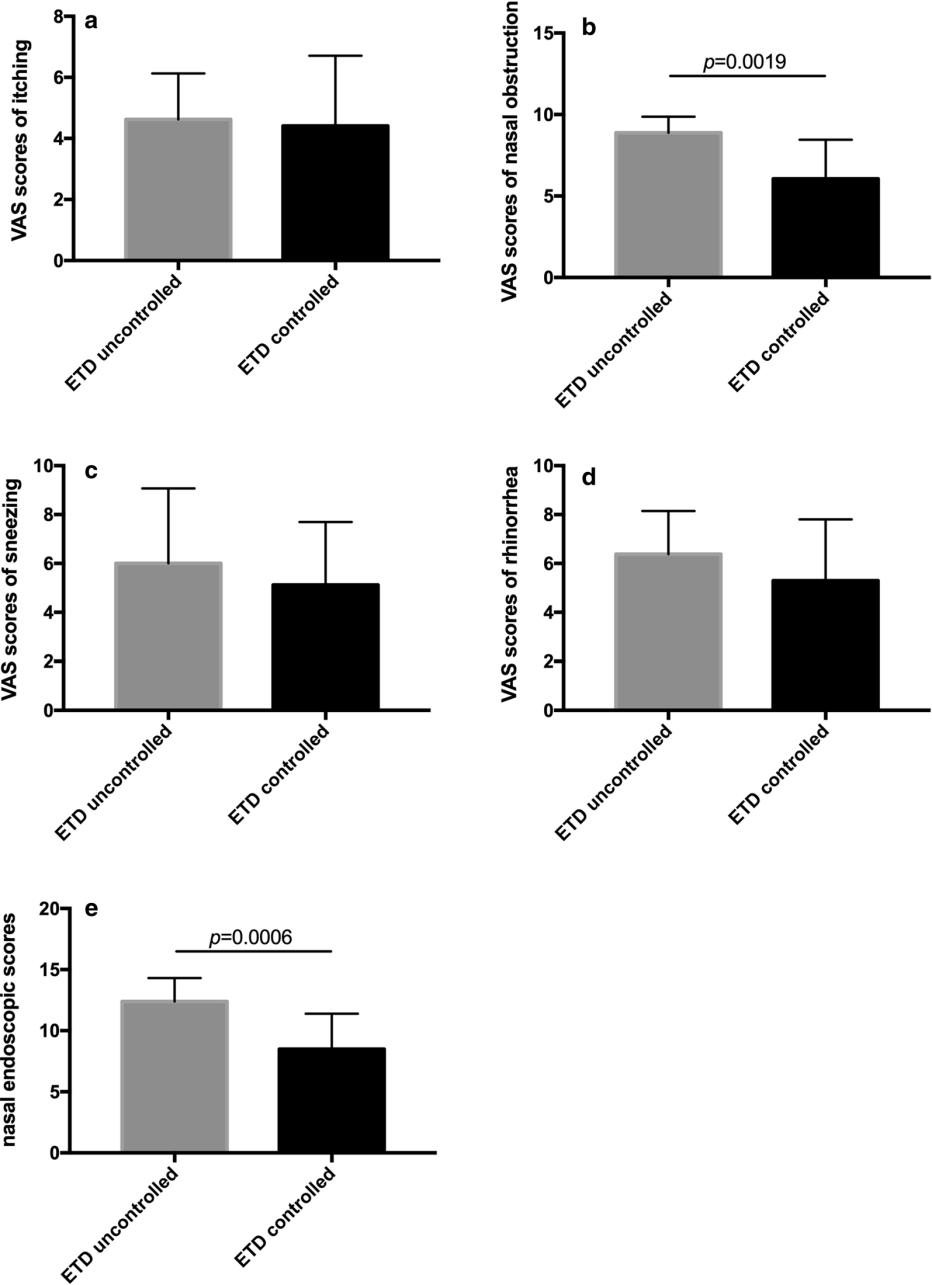
After 1 month of treatment in HDM-allergic rhinitis patients, improvements in nasal condition affect recovery from eustachian tube dysfunction (ETD). Nasal symptoms of itching (**a**), sneezing (**c**) and rhinorrhoea (**d**) were not significantly different between the controlled-ETD group (ETDQ-7 score < 15 after treatment) and the uncontrolled-ETD group (ETDQ-7 score ≥ 15 after treatment). The VAS scores for nasal obstruction (**b**) and the nasal endoscopic scores (**e**) were higher in the uncontrolled-ETD group than in the controlled-ETD group (*p *< 0.01)

To explore the reasons for the poor improvements in eustachian tube function in the 8 AR patients, we compared the nasal conditions after treatment between these 8 AR patients with ETDQ scores ≥ 15 (the uncontrolled-ETD group) and patients with ETDQ scores < 15 (the controlled-ETD group). We found that after 1 month of treatment, the nasal obstruction VAS scores (*p *= 0.0019) and nasal endoscopic scores (*p *= 0.0006) were significantly higher in the uncontrolled-ETD group. This suggests that uncontrolled AR symptoms, especially nasal obstruction, might contribute to irreversible ETD (Fig. 4).

## Discussion

ETD is a well-recognized comorbid manifestation of inflammatory diseases of the nasal cavity and nasal sinus [13]. However, due to the lack of appropriate ETD-specific assessment instruments, quantification of the prevalence of ETD had been limited before [20]. The TMM test was reported by Estève et al. [21] and has been widely used to evaluate eustachian tubes objectively [22]. The R value has a sensitivity of 0.91 in detecting the ET opening, but not specific [23]. Research showed that the interval correlation (ICC) was 0.68 for the TMM with 30 mbar, 0.62 for the TMM with 40 mbar and 0.61 for the TMM with 50 mbar in ETD patients, which means ET function change over time and TMM can only give a snapshot view on ET function [22]. The ETDQ-7 has been used as a validated patient-reported tool for assessment of the presence and severity of ETD symptoms since 2012. When 14.5 points are selected as the cut-off point, ETDQ-7 can provide 100% sensitivity and 100% specificity for classifying patients as ETD [19]. A recent study showed that the incidence of ETD in CRS patients is 48.5–68% [6, 7]. However, the prevalence of ETD in AR and the relationship between these conditions are still poorly understood.

Nasal VAS scores and ETDQ-7 scores were used to assess symptoms of the nose and eustachian tubes in our study. According to the subjective symptom scores, approximately 37.3% of AR patients had ETD, while up to 51.4% of moderate/severe AR patients had ETD. Among the four major symptoms of AR, nasal obstruction showed a moderate correlation with ETDQ-7 score. This means that more severe AR or a greater degree of nasal obstruction is more likely to cause ETD. Many patients with severe AR are prone to concomitant inferior turbinate hypertrophy [24] and torus tubarius swelling, causing both nasal obstruction and ETD [25]. Previous studies have shown that patients with nasal obstruction due to inferior turbinate enlargement or nasal septum deviation have significantly higher ETDQ-7 scores than healthy controls [26]. These results suggest that nasal obstruction is closely related to ETD. Furthermore, studies on nasal aerodynamics have shown that nasal resistance is significantly increased during nasal obstruction and that oral breathing can cause negative pressure in the nasopharynx and further negative pressure in the middle ear, which could aggravate ETD [27]. Similarly, nasal obstruction is also the most frequent nasal symptom in adult otitis media with effusion, which is considered closely related to ETD [28, 29].

Objective measures were also included in our study. Tympanograms, TMM test results and nasal endoscopic scores were taken for every AR patient. According to the TMM test results, 33.1% of AR patients had ETD, and 44.6% had severe AR combined with ETD. Nasal endoscopic scores for the pharyngeal orifices of eustachian tubes exhibited a strong correlation with ETDQ-7 scores. This suggests that the local conditions of the pharyngeal orifices of the eustachian tubes are closely related to ETD. Moreover, we found that the same patients could have different nasal endoscopic scores on the left and right sides. This could also explain why some AR patients only had unilateral ETD.

Our study also revealed that treatment with loratadine and mometasone furoate effectively improved nasal symptoms and significantly decreased ETDQ-7 scores and the frequency of abnormal TMM test results in AR patients. Both objective and subjective scores showed good consistency before and after treatment. Intranasal corticosteroids and antihistamines are recommended for treating perennial AR [30]. Although these medicines can also be applied for ETD, there is little evidence on effective medical treatments for ETD [31, 32]. Our study revealed that nasal steroids and antihistamines are efficient for ETD treatment in AR patients. However, it is worth noting that long-term use of these drugs may cause epistaxis, dry throat, and sedating antihistamines have been reported to cause seizures, sedation and gastrointestinal upset in children. Thus, it is important for a doctor to determine whether the potential efficacy of these treatments warrants the risk of side effects. In our study, only 2 patients had epistaxis and 5 patients had dry throat, any other side effects were not reported.

### Limitation

To our knowledge, this is the first pilot study on ETD in AR and to find that AR can cause ETD. However, there are some limitations. First, our study involved a referral system and a single centre; thus, the ETD cases may not reflect the epidemiology of ETD in all AR populations. Second, this study was not based on a double-blinded, randomized controlled trial. Third, although certain characteristics, such as smoking, laryngopharyngeal reflux and atopy, are not known to be risk factors for ETD, these factors were not considered in our analysis. Then, other nasal symptoms like itching, sneezing and rhinorrhoea showed very weak correlations with ETDQ-7; this may be caused by insufficient sample size. Finally, we used only oral antihistamines and nasal mometasone furoate for AR patients with ETD; other treatment approaches that can help to improve eustachian tube function, such as Buteyko breathing and Valsalva training, were not included in our study.

In conclusion, AR patients, especially those with severe nasal obstruction, are more likely to have ETD than individuals without AR. ETD in patients with AR is mainly characterized by ear blockage and ear pressure, and the appearance of symptoms is usually associated with the onset of rhinitis, which should attract attention in clinical work. Both ETDQ-7 scoring and TMM are effective means of assessing eustachian tube function in AR patients. Regular treatment for AR can reduce patients’ nasal symptoms and effectively improve eustachian tube function.

## Supplementary information

**Additional file 1. Table S1.** The degree of skin prick test. **Table S2.** The degree of specific immunoglobulin E level in serum. **Figure S1.** Specific immunoglobulin E levels to the 8 common allergens in AR patients. **Figure S2.** Treatment results of nasal symptom, ETDQ-7 and endoscopic scores in AR patients compared with control group. **Table S3.** The treatment results of eustachian tube dysfunction for AR patients.

## Data Availability

The data of this study are available from ResMan Clinical Trial Management Public Platform. However, restrictions that apply to the availability of these data, were used under license for the current study, and so are not publicly available yet. Data could be available from the corresponding author upon reasonable request.

## Abbreviations

AR: Allergic rhinitis
ARIA: Allergic rhinitis and its impact on asthma
CRS: Chronic rhinosinusitis
ETD: Eustachian tube dysfunction
ETDQ-7: Seven-item Eustachian Tube Dysfunction Questionnaire
HDM: House dust mite
RQLQ: Rhinoconjunctivitis quality of life questionnaire
TMM: Tubomanometry
VAS: Visual analogue scale
