# Supplementary material for: Eustachian tube dysfunction in patients with house dust mite-allergic rhinitis
Source: Clin Transl Allergy. 2020 Jul 15;10:30. doi: 10.1186/s13601-020-00328-9 (PMC7362452; doi:10.1186/s13601-020-00328-9)
Supplement: Supplementary file 1 — Additional file 1. Table S1. The degree of skin prick test. Table S2. The degree of specific immunoglobulin E level in serum. Figure S1. Specific immunoglobulin E levels to the 8 common allergens in AR patients. Figure S2. Treatment results of nasal symptom, ETDQ-7 and endoscopic scores in AR patients compared with control group. Table S3. The treatment results of eustachian tube dysfunction for AR patients. [file 13601_2020_328_MOESM1_ESM.docx]

Additional material

According to the patient's wishes, skin prick test (SPT) or specific IgE (sIgE) could be selected for allergen test. In our study, 41 patients chose the sIgE test and 18 patients chose SPT.

The SPT kit for house dust mite was from Wolwo Biotech, China. Negative (saline) and positive (histamine dihydrochloride solution) controls were included. The positive control must show a wheal diameter ≥ 3mm. A skin index (SI,$\mathrm{SI}=\frac{the wheal diameter of allergen}{the wheal dimeter of positive control}$) was used for the interpretation of results. The results are ranged from 0~++++ (Table S1).

Table S1 The degree of SPT

| Degree | 0(Negative) | + | ++ | +++ | ++++ |
| --- | --- | --- | --- | --- | --- |
| SI | 0 | <0.5 | 0.5≤SI<1 | 1≤SI<2 | ≥2 |

The serum level of specific immunoglobulin E (sIgE) to allergen was detected by ImmunoCAP assays. Our system contains 8 common allergens in southern China. The results could be divided into 6 degrees depending on the concentration (Table S2).

Table S2 The degree of sIgE

| Degree | 0(Negative) | 1 | 2 | 3 | 4 | 5 | 6 |
| --- | --- | --- | --- | --- | --- | --- | --- |
| sIgE(kUA/L) | <0.35 | 0.35~0.7 | 0.7~3.5 | 3.5~17.5 | 17.5~50 | 50~100 | >100 |

In our study, all the AR patients had an HDM-positive SPT result of ++ or above, or the level of sIgE to *Der p*1 showed 3 or above (>3.5 kUA/L). The multi-sensitized AR patients were also included with the sIgE level of *Der p*1 higher than any other allergens (Figure S1). However, the patients who had a positive allergen result but without symptoms were excluded.


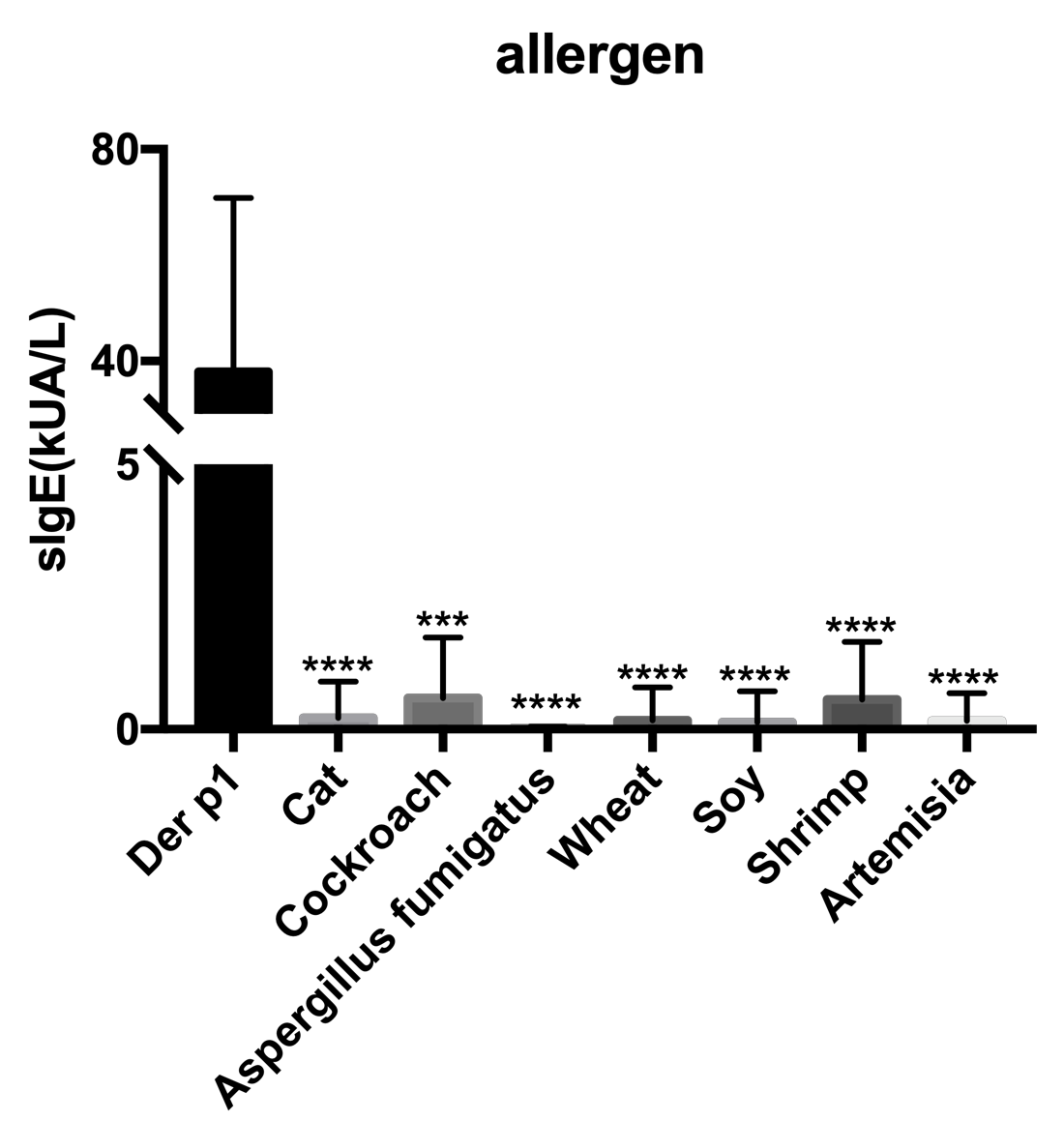


**Figure S1. Specific IgE levels to the 8 common allergens.** The average sIgE level of *Der p*1 was 38.010 ± 32.790 kUA/L, and significantly higher than any other allergens (cat 0.211 ± 0.689 kUA/L, cockroach 0.585 ± 1.147 kUA/L, aspergillus fumigatus 0.021 ± 0.026 kUA/L, wheat 0.170 ± 0.620 kUA/L, soy 0.136 ± 0.581 kUA/L, shrimp 0.558 ± 0.170 kUA/L, artemisia 0.160 ± 0.082 kUA/L) (***, *p*<0.001; ****, *p*<0.0001; compared with sIgE level of *Der p*1).

The therapeutic effect in AR patients

We have already evaluated the nasal condition and eustachian tube function before and after the treatment. In order to explore whether the treatment effect has reached a complete cure, we further compared the differences of the nasal condition and eustachian tube status in after treatment-AR patients to the control group. After 1 month treatment, the VAS scores for four major nasal symptoms and the nasal endoscopic scores were still significantly higher than control group (*p*<0.0001, Figure S2). However, the ETDQ-7 sores after treatment had no statistical difference with control group (Figure S2E). There was no statistical difference between 8 AR patients (13.6%) and 9 control subjects (15.3%)with an ETDQ-7 score ≥15 (χ^2^=0.069, *p*=0.7932) (Table S3). The number of AR patients with type A tympanograms (56 patients, 109 ears, 92.4%) increased after treatment, but still less than control group (59 subjects; 116 ears, 98.3%) (χ^2^=4.672, *p*=0.0307). The number of abnormal TMM was 10 patients (17 ears, 7.6%) in AR group after treatment, but still higher than 6 control subjects (7 ears, 5.9%) (χ^2^=4.638, *p*=0.0313) (Table S3).


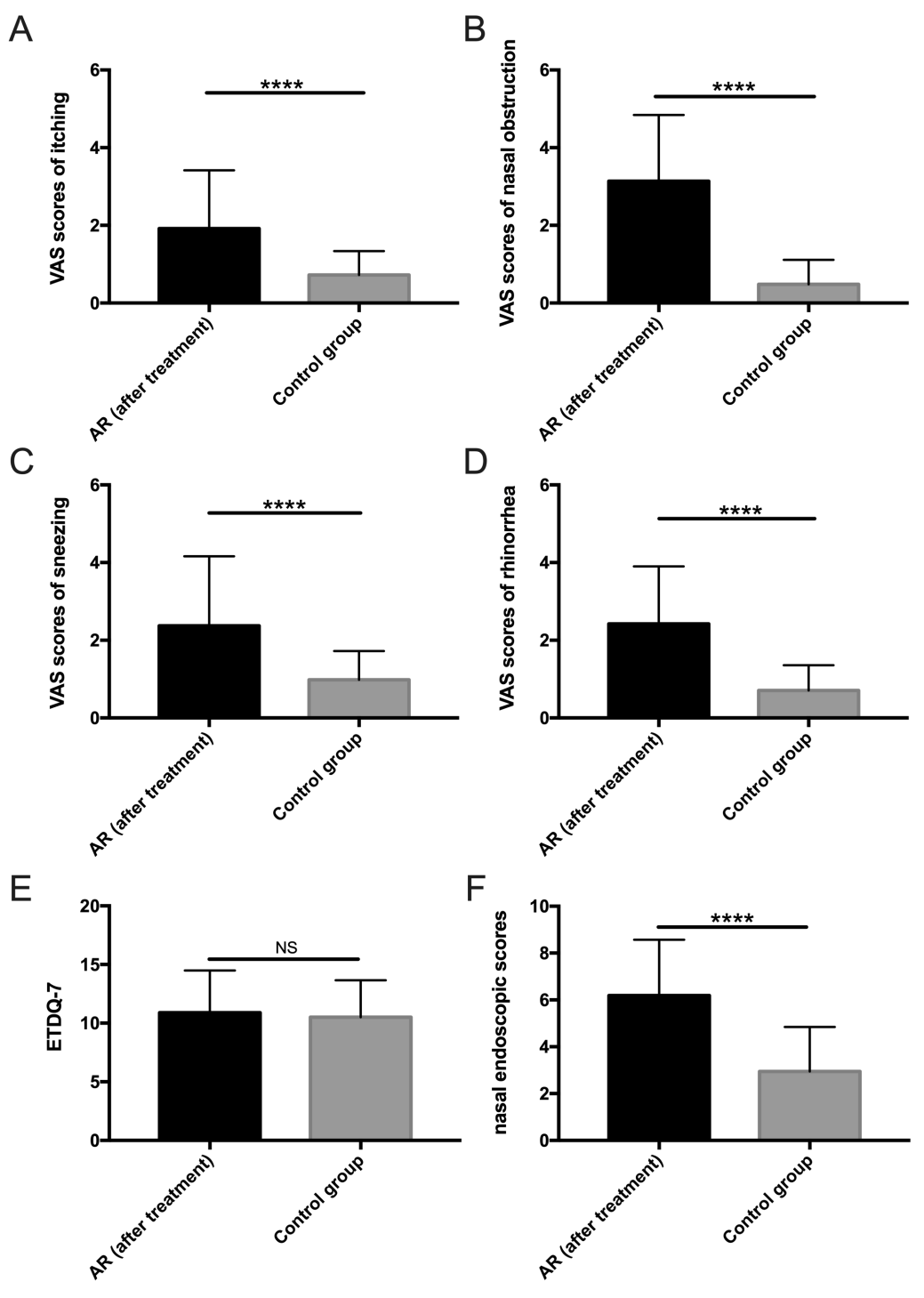


**Figure S2.** **Treatment results of nasal symptom, ETDQ-7 and endoscopic scores in AR patients compared with control group.** A-D. After one month of treatment, the VAS scores for itching(A), nasal obstruction(B), sneezing (C), rhinorrhea (D) in AR group were still higher than control group. E. After treatment, ETDQ-7 score in AR group has no statistical difference with control group. F. The nasal endoscopic scores in AR group was still higher after treatment, compared with the control group. (****, *p*<0.0001)

**Table S3**. The treatment results of eustachian tube for AR

|  | AR after treatment  (n=59) | Control group  (n=59) | χ^2^ | *p* |
| --- | --- | --- | --- | --- |
| Tympanometry ^1^ (236 ears) |  |  | 4.672 | *p*=0.0307 |
| Type A, N (%) | 109(92.4%) | 116 (98.3%) |  |  |
| Type B, N (%) | 0 | 0 |  |  |
| Type C, N (%) | 9 (7.6%) | 2 (1.7%) |  |  |
| TMM ^1^ (236 ears) |  |  | 4.638 | *p*=0.0313 |
| Normal, N (%) | 101(85.6%) | 111 (94.1%) |  |  |
| Abnormal, N (%) | 17(14.4%) | 7 (5.9%) |  |  |
| ETDQ-7 Score^2^  (118 patients) |  |  | 0.069 | *p*=0.7932 |
| <15, N (%) | 51(86.4%) | 50(84.7%) |  |  |
| ≥15, N (%) | 8(13.6%) | 9 (15.3%) |  |  |

1. Statistical analysis was performed by the number of ears.

2. Statistical analysis was performed by the number of patients.
